# Deviation of Chinese Adults’ Diet from the Chinese Food Pagoda 2016 and Its Association with Adiposity

**DOI:** 10.3390/nu9090995

**Published:** 2017-09-08

**Authors:** Xu Tian, Yingying Huang, Hui Wang

**Affiliations:** 1 College of Economics and Management, China Center for Food Security Studies, Nanjing Agricultural University, Nanjing 210095, China; xutian@njau.edu.cn; 2 College of Economics and Management, Nanjing Agricultural University, Nanjing 210095, China; 2015106010@njau.edu.cn; 3 Department of Epidemiology, School of Public Health, Nanjing Medical University, Nanjing 211166, China

**Keywords:** dietary status, Chinese dietary guidelines, China food pagoda, adiposity, adults

## Abstract

Changing diet in China contributes to a raising prevalence of overweight and obesity. This study aimed to evaluate the dietary status of Chinese adults (20–59 years old) using the China Food Pagoda (CFP) proposed in the Chinese Dietary Guidelines 2016 (CDG), and investigate the association between adiposity and deviation of real diet from CFP using an ordered logistic regression. Results showed that the consumption of fruits, eggs, meat, and poultry increased significantly during 2004–2011, while the consumption of cereal, potatoes, and beans dropped down significantly during the same period (all *p* < 0.05). Meanwhile, great disparity was detected between real consumption and recommended intake in CFP. In particular, a deficient intake was found for milk and milk products, eggs, and fruit, while over-consumption was observed for cereal, potatoes and beans, meat and poultry, legumes and nuts, oil, and salt. In addition, over-consumption of cereal, legumes and nuts, and salt, as well as under-consumption of vegetables, and meat and poultry, were associated with a higher risk of having high body mass index (BMI), while lower consumption of cereal, potatoes and beans, eggs, and higher consumption of vegetables contributed to low hazard of overweight/obesity (all *p* < 0.05). The huge disparity between real consumption and the CFP calls for specific health education campaigns.

## 1. Introduction

The food consumption pattern has been changing significantly in the past four decades in China, along with the rapid economic development [1]. In particular, Chinese consumers are switching from traditional Chinese food, which is characterized by grains and vegetables, to high-fat and high-sugar Western food [2,3,4]. Consumers’ food consumption patterns and dietary status are associated with regional economic development, resource exploitation and utilization, as well as cultural progress. They also reflect the living standards and health status of citizens [2,3,4]. In recent years, China has made great achievements in improving health and nutrition levels, but the rapid dietary change has led to raising concern on the coexistence of under-nutrition and over-nutrition, which are believed to contribute to the increasing prevalence of non-communicable diseases such as diabetes [4,5,6]. The China Nutrition and Chronic Disease Report 2015 revealed a rising threat from nutrition-related, non-communicable disease, and that the prevalence rate of obesity, cardia-cerebrovascular diseases, diabetes, and cancer increased significantly in the past years [7]. 

In order to improve the health status of Chinese citizens, the National Health and Family Planning Commission of the People’s Republic of China (NHFPC) released the Chinese Dietary Guidelines 2016 (CDG 2016) based on basic nutrition demands and health conditions [8]. This research evaluated the dietary status of Chinese adults between 2004 and 2011 using the recommended consumption level of the Chinese Food Pagoda (CFP) 2016, the quantitative dietary guideline presented in CDG 2016. In addition, the association between dietary deviation and adiposity was also investigated using an ordered logistic regression model. Uncovering the difference between China’s real food consumption and the recommended level in CFP 2016, and its association with body mass, could contribute to a more effective guidance on peoples’ food consumption habits, which also has strong policy implications for alleviating the public burden caused by malnutrition and chronic diseases.

## 2. Materials and Methods

### 2.1. Study Subjects

The recent four waves (2004, 2006, 2009, and 2011) of China Health and Nutrition Survey (CHNS) data were adopted in this research. The CHNS data is jointly collected by the Carolina Population Center at the University of North Carolina at Chapel Hill and the National Institute for Nutrition and Health at the Chinese Center for Disease Control and Prevention. The CHNS is an ongoing cohort survey on approximately 4000 families each year in both urban and rural areas in nine provinces of China (Guangxi, Guizhou, Henan, Heilongjiang, Hubei, Hunan, Jiangsu, Liaoning, and Shandong; three municipalities of Beijing, Chongqing, and Shanghai were included in 2011), which are located in different regions of the country, including the north and south area, well-developed east-coastal region, and poor remote region. Samples were selected by utilizing a multi-stage, random cluster strategy, and can be treated as representative of the Chinese population. More detailed information can be found elsewhere [9].

A total of 49,966 observed samples were obtained from food consumption in the recent four waves data. After merging with data of personal characteristics, 22,371 were removed due to incomplete information, leaving 27,595 observations. We only focused on adults aged between 20 and 59 years old (*n* = 17,652) because children and old people have very alternative dietary recommendations. Observations whose daily energy intake is smaller than 520 kilocalories (minimum energy needed to survive) and greater than 8000 kilocalories (about three time the mean calorie intake) were removed to reduce measurement error (*n* = 27). We further removed pregnant (*n* = 157) women and respondents who had been sick or injured in the last four weeks (*n* = 2180), as their food consumption during the survey is not representative. In addition, 836 more observations were lost due to unrealistically low BMI (<15) or high BMI (>50). Finally, 14,452 individuals remained in our sample, of which 48.1% are male (*n* = 6949), and 51.9% are female (*n* = 7503). Urban residents account for 31.9% of the total population (*n* = 4610), and rural residents account for the rest 68.1% (*n* = 9842). The samples collected from 2004, 2006, 2009, and 2011 are 3427 (23.7%), 3369 (23.3%), 3454 (23.9%), and 4202 (29.1%), respectively. Finally, the number of young adults (aged between 20 and 39) and older adults (aged between 40 and 59) are 5296 (36.6%) and 9156 (63.4%), respectively. 

### 2.2. Assessment of Food Consumption

The CHNS records the consumption quantity of each of the food items listed in the China Food Composition Table [10,11], including all food consumed at home and away from home, for each individual using the 24-h recall method, for three consecutive days within one week. The individual food recall data was adopted in our research to measure food consumption structure. In addition, household food consumption was also recorded by calculating the changes in the home food inventory for the same three consecutive days. All foods and condiments were carefully recorded and measured at the beginning and end of the 3-day survey period, no matter if they were purchased from markets or picked from the participants’ own gardens. Meanwhile, the number of meals eaten at home during the survey days is also collected for all family members, which was used to generate the food consumption per person per day. Family food inventory data was used to calculate individual consumption of oil and salt, which was not available in individual food recall data. All food data were recorded by trained interviewers through face-to-face interviews by using food models and pictures, and included the types, amounts, and locations of consumption of all food items consumed. More details of the CHNS data can be found in previous literature [9,12]. 

### 2.3. Measurement of Adiposity

Adiposity was measured by body mass index (BMI), which was calculated by dividing the weight (kg) by the square of the height (m^2^) of each participant. It was further divided into four categorical levels based on the criteria recommended by Working Group on Obesity in China [13], namely underweight (BMI < 18.5 kg·m^−2^), normal (18.5 ≤ BMI < 24 kg·m^−2^), overweight (24 ≤ BMI < 28 kg·m^−2^), and obesity (BMI ≥ 28 kg·m^−2^).

### 2.4. Measurement of Other Covariates

Household net income per capita was adopted as a measure of income. The physical activity levels of adults were measured according to the occupation type, and ranged from 1 to 5: namely, 1 = very light physical activity, working in a sitting position (for example, office worker or watch repairer); 2 = light physical activity, working in a standing position (for example, sales person or teacher); 3 = moderate physical activity (for example, student or driver); 4 = heavy physical activity (for example, farmer or dancer); and 5 = very heavy physical activity (for example, loader, logger, or miner). We further classified 1 and 2 as light activity, 3 as medium activity, 4 and 5 as heavy activity [2,3,4]. Smoking was defined as whether respondents were currently smoking (0 = currently not smoking, 1 = currently smoking), and drinking was defined as whether respondents drank in the past year (0 = did not drink in the past year, 1 = drank alcohol in the past year); urbanization is defined by a multidimensional 12-component urbanization index, which captures the population density, physical, social, cultural, and economic environment [6].

### 2.5. China Food Pagoda 2016

The CDG 2016 was jointly created by the Chinese Center for Disease Control and Prevention (CDC), National Health and Family Planning Commission of the People’s Republic of China, and the Chinese Nutrition Society. These two institutions take the main responsibility of developing, promoting, and implementing nutrition and nutrition-related health policies in China. The CDG 2016 recommended a food-based dietary guideline under several key principles, including nutrition balance, food diversity, preventing malnutrition-related non-communicable disease. The standard daily diet for healthy adults with lower and upper bounds was named as the CFP 2016. In addition, the CDG 2016 also presents specific dietary guidelines for infant, children, adolescent, pregnant women, breastfeeding women, and old people. We employ the CFP 2016 to evaluate Chinese people’s dietary status because our research only focuses on adults aged between 20 and 59. The CFP presents the main principles of CDG 2016 in a figure, and transforms the principle into recommended daily consumption quantity for five food groups: (1) cereal potato and beans; (2) fruits and vegetables; (3) animal products (eggs, aquatic products, meat and poultry); (4) legumes and nuts, milk and its products; (5) oil and salt. A specific value was set up for 10 individual food items as a reference level. More detailed values were presented in the last column of Supplementary Table S1. Both minimum and maximum consumption quantities were set up for cereal, potato and beans, fruits, vegetables, eggs, aquatic products, meat and poultry, legumes and nuts, milk and its products, and oil, which were defined as lower and upper bounds, respectively. Differently from this, only the minimum consumption level was proposed for milk and its production, while only the maximum consumption quantity was recommended for salt. We thus define these two values as the lower and upper bounds, respectively. Under-consumption was defined as when the real consumption was lower than the lower bound of CFP 2016, and over-consumption was defined as when the real consumption was higher than the upper bound [14].

### 2.6. Statistical Methods

Trend test was conducted by regressing food consumption on time. One-side *t* test was adopted to compare the mean food consumption level between different population groups (urban vs. rural, young adult vs. older adult, male vs. female). Ordered logistic regression was adopted to estimate the odds ratio of the risk of falling into higher weight categories (1 = underweight, 2 = normal weight, 3 = overweight, 4 = obesity). Significance level was set at *p* < 0.05. All statistics were performed in stata (version 13; Stata Corporation, College Station, TX, USA).

## 3. Results

### 3.1. Summary of Food Consumption and Covariate by BMI Group

Table 1 presented the mean and standard deviation of the ten food categories listed in the CFP, and the social-economic status. Different dietary patterns were observed for people in different BMI groups. However, great heterogeneity was also detected in covariates. Therefore, investigating the association between dietary pattern and adiposity should be conducted after adjusting the covariates.

### 3.2. Overall Evaluation of Dietary Status for Chinese Adults

The real food consumption and the recommended food consumption levels in CFP 2016 were presented in Supplementary Table S1. Results showed that the mean consumption of cereal, potatoes and beans, meat and poultry, legumes and nuts, oil, as well as salt, were significantly higher than the upper bound of CFP 2016 (all *p* < 0.05). In particular, more than 50% of people over-consumed oil and salt. To the contrary, the mean consumption of fruits, eggs, aquatic products, as well as milk and its products were significantly lower than the lower bound of CFP 2016 (all *p* < 0.05), of which more than 90% percent of people under-consumed fruits and milk and its products. The only food group whose mean consumption fell into the recommended boundary is vegetables. 

**Figure 1:**
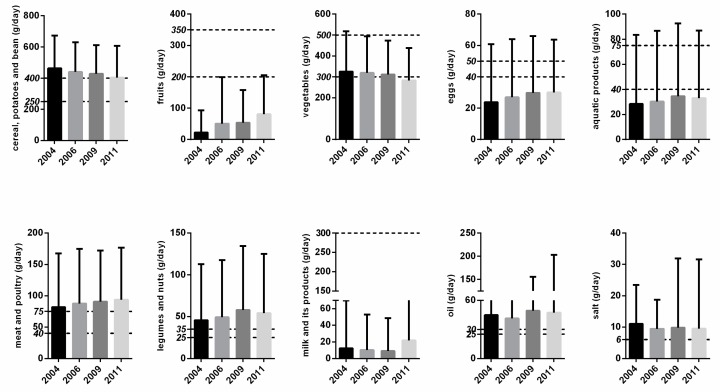
Consumption of individual food groups from 2004 to 2011. Note: The horizontal dashed line refers to the recommended consumption level. The upper line refers to the upper bound, while the lower line refers to the lower bound. The lower and upper bounds for cereal, potato and beans, fruits, vegetables, eggs, aquatic products, meat and poultry, legumes and nuts, milk and its products, and oil are 250/400, 200/350, 300/500, 40/50, 40/75, 40/75, 25/35, 25/30, respectively. Only one standard was set for milk and salt in the Chinese Food Pagoda, which are 300 (lower bound) and 6 (upper bound), respectively. The bar refers to the mean consumption, and the solid black short line above the bar refers to mean ± standard deviation.

Figure 1 presents the yearly mean food consumption for four survey years (2004, 2006, 2009, 2011). An increasing trend was detected for fruits, eggs, and, meat and poultry, while a descending trend was found for cereal, potatoes and beans (all *p* < 0.05), and a similar declining trend was also detected for vegetables (*p* = 0.076). The consumption of milk and its products in 2011 was higher than that in the previous years, while the consumption of salt decreased after 2004. Compared with CFP 2016, the mean consumption of cereal, potatoes, and beans was converging to the recommend boundary, while the consumption of vegetables declined and dropped out of the lower bound in 2011.

### 3.3. Dietary Status of Different Subpopulation

#### 3.3.1. Urban and Rural

Table 2 presents the mean consumption for urban and rural populations, respectively. Urban residences had significantly higher consumption of fruits, eggs, aquatic products, meat and poultry, as well as milk and its products, than their rural counterparts, but their consumption of cereal, potatoes and beans, and vegetables were lower than that of rural residence (all *p* < 0.05). Comparing to the recommended level in CFP 2016, meat and poultry, legumes and nuts, oil, as well as salt, were over-consumed in both regions, while fruits, eggs, and milk and its products, were under-consumed in both regions. In addition, the mean consumption of cereal, potatoes and beans in rural area was higher than the upper bound of CFP 2016, while the mean consumption of aquatic products in rural areas and the mean consumption of vegetables in urban areas were lower than the lower bound. 

#### 3.3.2. Age Group

The dietary statuses of young adults (aged between 20 and 39) and older adults (aged between 40 and 59) were also shown in Table 2. Older adults had a significantly higher consumption of vegetables, aquatic products and salt, but lower consumption of meat and poultry (all *p* < 0.05). However, the differences were very small in magnitude, and the dietary statuses of both groups were consistent with the whole population. 

#### 3.3.3. Male and Female

Male respondents had significantly higher consumption of cereal, potatoes and beans, vegetables, eggs, aquatic products, meat and poultry, as well as legumes and nuts, than female respondents (all *p* < 0.05), while their consumption of fruits, and milk and its products, were significantly lower than female counterparts (all *p* < 0.05). Meanwhile, the dietary status of males and females evaluated by CFP 2016 were similar, in that fruits, eggs, aquatic products, and milk and its products were under-consumed, while meat and poultry, legumes and nuts, oil, and salt, were over-consumed. In addition, male respondents slightly over-consumed cereal, potatoes and beans, while female respondents slightly under-consumed vegetables. 

### 3.4. Trend of Adiposity

**Figure 2:**
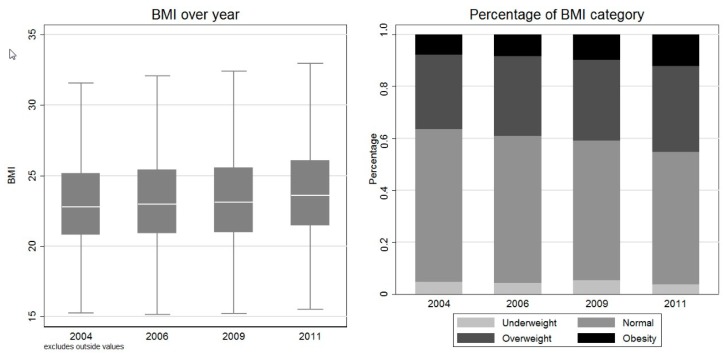
Changing BMI over year. Note: The box in the left panel is the 75% and 25% percentiles, and the white line within the box is the median value. Two gray horizontal lines refer to the upper and lower adjacent values. The upper adjacent value = 75th percentile + 1.5 × (75th percentile − 25th percentile), and the lower adjacent value = 25th percentile − 1.5 × (75th percentile - 25th percentile). Outside values, which are defined as those greater than the upper adjacent value or smaller than the lower adjacent value, are excluded from the box graph.

Figure 2 presented the trend of BMI and its category in four waves. The box graph in the left panel showed that the mean BMI increased steadily over years. The histogram in the right panel revealed that percentage of respondents with normal BMI decreased from 58.91% in 2004 to 51.09% in 2011, while proportions of overweight and obese people increased from 28.63% and 7.79% in 2004, to 33.06% and 12.11% in 2011, respectively.

### 3.5. Association between Adiposity and Dietary Deviation from CFP 2016

Table 3 reported the association between adiposity and dietary deviation from CFP 2016 using ordered logistic regression. Deviation from CFP 2016 was associated with the risk of having higher BMI values for 5 of 10 food categories. In particular, over-consumption of cereal, potatoes and beans, and salt were associated with higher risk of falling into higher weight categories, but under-consumption of cereal, potatoes and beans, and eggs contributed to a lower risk of having greater BMI (all *p* < 0.05). In addition, over-consumption of vegetables lowered the risk of getting high BMI values, while under-consumption of meat increased the likelihood of having more adiposity (all *p* < 0.05). 

## 4. Discussion

This study employed the CFP 2016 to evaluate the dietary status of Chinese adults in 2004, 2006, 2009, and 2011. Results showed that fruits, eggs, and milk and its products, were under-consumed for most Chinese adults, while cereal, potatoes and beans, meat and poultry, legumes and nuts, oil, and salt were over-consumed for most adults. Moreover, we also found that the consumption of fruits, eggs, and meat and poultry were increasing, while the consumption of cereal, potatoes and beans, and vegetables were decreasing. In addition, an increasing trend of BMI was also detected for Chinese adults. We finally employed an ordered logistic model to investigate the association between dietary deviation and weight category. Over-consumption of cereal, potatoes and beans, and salt, as well as under-consumption of meat, increased the likelihood of having higher body mass, while under-consumption of cereal, potatoes and beans, and eggs, and over-consumption of vegetables played a protective role forgetting high BMI values.

In general, Chinese adults consumed too little fruits, eggs, and milk and its products, but too much cereal, potatoes and beans, meat and poultry, legumes and nuts, oil, and salt. In particular, the consumption of oil, and meat and poultry, followed an increasing trend, which might further led to a higher risk of some chronic diseases, such as obesity, hyperlipidemia, and atherosclerosis [15]. Current literature has already found that rich Chinese people tend to have a more high-valued and diversified diet, consisting of fruits, aquatic products, milk and its products, but less high calorie dense foods, such as cereals [2,3,4]. Along with the rapid income growth, we can expect that the demand for fruits, aquatic products, milk and its products will keep rising. Meanwhile, the direct consumption level of cereals will drop, but the demand for feed cereals will go up, due to a soaring demand for animal food such as meat, poultry, and aquatic products. Therefore, the total demand for cereals will keep rising in the near future because more cereals are needed to feed animals [16,17,18,19]. The surging demand for meat and poultry is at the expense of a deteriorating environment, such as increasing gaseous emissions and nonpoint pollution. Therefore, controlling the consumption of meat and poultry can not only reduce the risk of overweight and obesity, but also benefit the environment. 

Comparisons between different subsamples indicated that the dietary status of urban residences were better than their rural counterparts. The difference might be caused by the higher income and food expenditure in urban families [20], so that they can buy more expensive foods, such as fruits, aquatic products, eggs, as well as milk and its products [4]. Moreover, urban adults were well-educated, thus, they might know more about nutrition and health. Furthermore, urban regions had higher food availability [4], and urban adults were more likely to work in sectors with lower physical activity, thus they had lower demand for high-calorie density food, such as cereal, potatoes, and beans [21]. On the contrary, rural residences were mainly involved in heavy-activity jobs, and preferred cheap high-calorie density food, and home-grown cereals and vegetables [21]. The regional dietary difference also reflected the nutrition transition pattern, along with economic development [22]. Our findings were consistent with the nutrition transition pattern found in previous studies, that Chinese people were shifting from a cereal-based traditional diet to a more diversified diet characterized by increasing consumption of high value foods, such as animal products, milk and its products, as well as fruits [1,21,23]. Our results also found that the dietary status of older adults was much closer to the recommended consumption in CFP 2016 than that of young adults. One possible reason is that older adults care more about the healthy impacts of food consumption, and they also had relatively more free time and higher financial capability to improve their diet quality [4,24].

The association study represented that the over-intake of cereal was positively associated with higher weight categories, while deficient consumption of cereal contributed to a lower risk of having high BMI values. Previous studies also indicated that refined grain and wheat, which comprised up to 90% of the staple food in the CDG 2016, were strongly associated with obesity [25,26]. The associations between intake of vegetables with adiposity were concordant with previous studies [27]. Over consumption of vegetables would reduce the risk of adiposity. Intriguingly, insufficient consumption of egg acted as a protective factor for adiposity, while deficient consumption of meat and poultry increased the hazard of overweight/obesity. One possible reason is that meat and poultry were difficult to prepare at home, and usually consumed in restaurants. In particular, poor people who cannot afford meat in their daily diet, might only have the chance to eat meat and poultry in hosted meals during festivals or celebrations in restaurants [6]. Foods prepared in restaurants are usually oily, and the food survey cannot capture the oil used in preparing food away-from-home, thus, the recorded energy intake of poor people might be underestimated. After adjusting for total energy intake in the ordered logistic regression, poor people who eat insufficient meat might have higher real energy intake than others, thus, they were more likely to get higher BMI. On the contrary, egg could be easily cooked at home and served as the main protein source for many Chinese people. Therefore, under-consumption of egg played a protective role of overweight/obesity. Another finding worth noting is that over-consumption of salts was significantly correlated with higher weight categories, which is in line with previous documents [28]. 

Several limitations should be mentioned in the present study. First, this study was a cross-sectional study, and the association between dietary deviation and adiposity cannot be taken simply as causality. Second, the various functions of different food categories on adiposity need further clinical studies. Third, specific guidelines for infants, children, adolescents, pregnant women, breastfeeding women, and old people, are also set in the CDG 2016, but our research only focused on adults aged between 20 and 59, thus, our results are only applicable to adults. Fourth, dietary data was measured using 3-day recall data, which were used as a proxy for the dietary habits of respondents. However, individuals’ diets might change over time (e.g., Chinese people tend to eat more meat and aquatic products in the winter, particularly during spring festival), and the 3-day recall might record the consumption of some food as zero, if they are accidently not consumed during the survey period. Thus, we call for more representative dietary surveys in different seasons over the year. In addition, several limitations of the CFP 2016 should also be mentioned: (1) CFP 2016 only provided a rather crude dietary guideline using broad food categories, while different food items play very different roles in health. For instance, oils high in saturated fats are associated with higher risk of cardiovascular disease, but those that contain predominantly polyunsaturated fats are considered healthy; (2) Some principles in CDG 2016, such as guidelines for sugar, are not presented in the CFP 2016, and thus, we did not take it into account in this study; (3) The requirement and preference for daily diet vary greatly across adults with different gender, body size, and age. For example, vegetarians can eat a balanced and healthy diet without meat or poultry, and females may prefer a diet with less oil and salt. Therefore, specific dietary guidelines should be provided for heterogeneous individuals. Despite those limitations, several strengths were worth mentioning as well. First, according to our knowledge, this is the first paper evaluating Chinese residents’ dietary pattern using CFP 2016. Second, we compared the dietary pattern of different sub-populations with the reference in CFP 2016, which proposed several potential targeted strategies for each sub-population. Third, the deviation of real diet from recommended diet and adiposity was also investigated.

## 5. Conclusions

In conclusion, our study indicated that the dietary status of Chinese adults was still far away from the standard noted in the CFP 2016. In particular, the consumption of fruit, and milk and its products, were far below the recommended level, while oil and salt were already over-consumed for most people. Therefore, a diet with more fruit, milk and its products, but less oil and salt, should be promoted in the future, which might also contribute to a better health status of Chinese citizens [21]. In addition, over-consumption of cereal, legumes and nuts, and salt, as well as under-consumption of vegetables, and meat and poultry, increased the risk of having higher BMI, while deficient consumption of cereal, potatoes and beans, eggs, and high consumption of vegetables was associated with reduced risk of overweight/obesity.

## Figures and Tables

**Table 1:** Characteristics of participants.

Variable	Total (*n* = 14,452)	Underweight (*n* = 646)	Normal (*n* = 7933)	Overweight (*n* = 4477)	Obesity (*n* = 1396)
Food category (g/day)					
Cereal potato and beans	432.7 ± 198.1 ^a^	418.3 ± 193.5	431.0 ± 200.3	434.5 ± 191.1	443.4 ± 208.7
Fruits	53.3 ± 117.7	52.8 ± 114.2	49.9 ± 113.9	58.9 ± 126.7	55.7 ± 109.6
Vegetables	307.9 ± 172.1	311.0 ± 163.4	310.6 ± 173.8	305.6 ± 170.0	298.3 ± 172.4
Eggs	27.8 ± 36.0	23.1 ± 29.5	26.2 ± 36.5	30.4 ± 36.1	30.5 ± 34.4
Aquatic products	31.6 ± 55.9	32.8 ± 56.8	31.4 ± 57.2	32.4 ± 53.5	29.9 ± 55.5
Meat and poultry	88.8 ± 84.3	96.3 ± 92.0	87.8 ± 82.5	91.0 ± 86.8	83.4 ± 82.6
Legumes and nuts	51.9 ± 71.0	50.6 ± 61..0	49.3 ± 68.9	54.9 ± 73.3	57.7 ± 78.6
Milk and its products	13.7 ± 52.8	16.1 ± 58.8	13.3 ± 52.8	14.8 ± 53.4	11.5 ± 47.1
Oil	45.8 ± 102.6	43.0 ± 41.4	46.5 ± 128.4	44.3 ± 52.8	47.9 ± 74.2
Salt	9.9 ± 17.8	9.1 ± 11.7	9.7 ± 18.6	10.3 ± 15.0	10.6 ± 23.2
Socio-economic variables					
Income (Yuan/year/capita)	18,740.4 ± 25,233.7	17,982.5 ± 23,580.7	18,084.0 ± 24,046.0	19,551.8 ± 26,710.1	20,218.8 ± 27,480.0
Household size	1.8 ± 0.8	1.9 ± 0.9	1.8 ± 0.8	1.8 ± 0.7	1.8 ± 0.8
Energy(Kcal)	2156.5 ± 680.7	2109.6 ± 633.9	2150.8 ± 678.4	2174.0 ± 677.5	2154.6 ± 710.1
Physical activity level ^b^	3.7 ± 1.2	3.8 ± 1.2	3.8 ± 1.2	3.6 ± 1.2	3.6 ± 1.2
Age(year)	42.8 ± 10.3	37.8 ± 11.9	41.8 ± 10.5	44.8 ± 9.4	44.4 ± 9.4
Male (%) ^c^	48%	42%	47%	50%	49%
Smoking (%)	29%	26%	30%	29%	27%
Ever drink (%)	36%	30%	34%	38%	39%
Urbanization Index	66.6 ± 20.0	66.1 ± 19.9	65.5 ± 20.2	68.0 ± 19.9	68.0 ± 19.1

Notes: ^a^ all values presented as mean ± SD; ^b^ physical activity level of adults were measured according to the occupation type, and ranged from 1 to 5: namely, 1 = very light physical activity, working in a sitting position; 2 = light physical activity, working in a standing position; 3 = moderate physical activity; 4 = heavy physical activity; and 5 = very heavy physical activity. We further classified 1 and 2 as light activity, 3 as medium activity, 4 and 5 as heavy activity; ^c^ % in “Total” section refers to percentage of subsample in total sample, while in body mass index (BMI) subcategories refer to percentage in the subcategories.

**Table 2:** Daily food intakes (g/day) of people by region, age and gender in China.

Food Group	Region		Age		Gender		Dietary
	Urban	Rural	20–39 years	40–59 years	Male	Female	Guidelines
Cereal potato and beans	378.8 ± 178.0 ^a^	459.8 ± 202.1 *	434.4 ± 202.5	431.7 ± 195.5	470.8 ± 205.2	397.4 ± 184.3 *	250–400
Fruits	72.3 ± 130.9	43.8 ± 109.2 *	53.9 ± 116.5	53 ± 118.4	48.0 ± 114.7	58.3 ± 120.1 *	200–350
Vegetables	296.7 ± 161.8	313.5 ± 176.7 *	297.1 ± 163.4	314.2 ± 176.6 *	321.6 ± 181.5	295.2 ± 161.8 *	300–500
Eggs	31.3 ± 37.7	26.0 ± 34.9 *	27.1 ± 36.6	28.1 ± 35.6	28.5 ± 36.1	27.1 ± 35.9 *	40–50
Aquatic products	40.8 ± 63.0	27.0 ± 51.3 *	30.5 ± 53.0	32.5 ± 57.4 *	34.1 ± 59.7	29.3 ± 52.0 *	40–75
Meat and poultry	111.1 ± 88.8	77.5 ± 79.6 *	91.7 ± 87.4	87.1 ± 82.4 *	99.2 ± 90.3	79.1 ± 77.1 *	40–75
Legumes and nuts	58.7 ± 73.4	48.5 ± 69.6 *	50.7 ± 69.7	52.6 ± 71.8	55.1 ± 74.3	49.0 ± 67.7 *	25–35
Milk and its products	30.9 ± 72.1	5.1 ± 36.7 *	13.0 ± 47.1	14.2 ± 55.8	11.8 ± 45.8	15.5 ± 58.4 *	>300
Oil	45.9 ± 60.5	45.7 ± 118.2	43.6 ± 107.2	47.0 ± 99.8	46.2 ± 82.8	45.4 ± 118.0	25–30
Salt	9.6 ± 15.7	10.1 ± 18.8	9.1 ± 13.4	10.4 ± 19.9 *	10.0 ± 18.2	9.9 ± 17.5	<6

Notes: ^a^ Values are presented in mean ± standard deviation; * Significantly different from the mean of another group within subgroups at *p* < 0.05.

**Table 3:** Association between weight category and the deviation of food consumption from CFP ^a^.

Deviation	Weight Category ^b^
Low cereal	0.813 (0.728, 0.908) *^,c^
Higher cereal	1.132 (1.045, 1.226) *
Low fruit	0.945 (0.824, 1.083)
Higher fruit	1.066 (0.851, 1.337)
Low vegetable	1.070 (0.993, 1.153)
Higher vegetable	0.883 (0.783, 0.996) *
Low egg	0.859 (0.772, 0.956) *
Higher egg	1.083 (0.958, 1.224)
Low fish	1.054 (0.955, 1.164)
Higher fish	1.013 (0.899, 1.140)
Low meat	1.141 (1.033, 1.261) *
Higher meat	0.971 (0.885, 1.066)
Low nut	0.974 (0.873, 1.087)
Higher nut	1.045 (0.934, 1.169)
Low milk	0.960 (0.555, 1.661)
Low oil	1.002 (0.889, 1.129)
Higher oil	0.995 (0.885, 1.119)
Higher salt	1.081 (1.007, 1.162) *

Notes: ^a^ Association was investigated by ordered logistic regression after adjusted by household income per capita, household size, total energy intake in logarithm, physical activity level, age, gender, smoking (0 = currently not smoking, 1 = currently smoking) and drinking (0 = did not drink in the past year, 1 = drank alcohol in the past year) status, and urbanization index (defined by a multidimensional 12-component urbanization index, which captures the population density, physical, social, cultural, and economic environment). Physical activity is defined based on occupation (1 = very light physical activity, working in a sitting position; 2 = light physical activity, working in a standing position; 3 = moderate physical activity; 4 = heavy physical activity; and 5 = very heavy physical activity); ^b^ Weight category is defined as: 1 = underweight (BMI ≤ 18.5), 2 = normal weight (BMI ≥ 18.5 & BMI < 24), 3 = overweight (BMI ≥ 24 and BMI < 28), 4 = obesity (BMI ≥ 28) is defined as ^c^ Values are odds ratios, and values in brackets are 95% CI; * Statistically significant at *p* < 5%.

## Supplementary Materials

The following are available online at www.mdpi.com/2072-6643/9/9/995/s1, Table S1: Daily food consumption (g/d) in China.
