# Supplementary material for: Deviation of Chinese Adults’ Diet from the Chinese Food Pagoda 2016 and Its Association with Adiposity
Source: Nutrients. 2017 Sep 8;9(9):995. doi: 10.3390/nu9090995 (PMC5622755; doi:10.3390/nu9090995)
Supplement: Supplementary file 1 [file nutrients-09-00995-s001.zip › nutrients-214053-supplementary.pdf]

# Supplementary Materials: Deviation of Chinese Adults' Diet from the Chinese Food Pagoda 2016 and Its Association with Adiposity

**Xu Tian, Yingying Huang and Hui Wang**

**Table S1 Daily food consumption (g/d) in China**

| Food group | Real Consumption | | | | | Trend Test ($p$) | Dietary Guidelines | | | |
|---|---|---|---|---|---|---|---|---|---|---|
| | Total | 2004 ($n$ = 3427) | 2006 ($n$ = 3369) | 2009 ($n$ = 3454) | 2011 ($n$ = 4202) | | Recommended | Under | Within | Above |
| Cereal potato and beans | 432.7 ± 198.1 [a*] | 463.8 ± 209.5 | 440.7 ± 190.0 | 428.7 ± 183.2 | 404.2 ± 202.4 | 0.008 | 250–400 | 13% [b] | 39% | 48% |
| Fruits | 53.3 ± 117.7 [#] | 22.1 ± 79.8 | 50.9 ± 147.8 | 53.5 ± 104.4 | 80.6 ± 124.6 | 0.038 | 200–350 | 90% | 7% | 3% |
| Vegetables | 307.9 ± 172.1 | 324.3 ± 193.4 | 319.1 ± 175.3 | 311.2 ± 162.6 | 282.9 ± 155.0 | 0.076 | 300–500 | 54% | 35% | 11% |
| Eggs | 27.8 ± 36.0 [#] | 23.8 ± 37.0 | 27.0 ± 37.1 | 29.8 ± 36.3 | 30.0 ± 33.7 | 0.050 | 40–50 | 70% | 11% | 19% |
| Aquatic products | 31.6 ± 55.9 [#] | 28.4 ± 55.1 | 30.2 ± 56.4 | 34.5 ± 58.1 | 33.0 ± 54.0 | 0.148 | 40–75 | 71% | 13% | 16% |
| Meat and poultry | 88.8 ± 84.3 [*] | 81.8 ± 85.6 | 87.7 ± 87.0 | 90.7 ± 81.2 | 93.7 ± 83.2 | 0.016 | 40–75 | 33% | 20% | 47% |
| Legumes and nuts | 51.9 ± 71.0 [*] | 45.7 ± 67.1 | 49.3 ± 68.4 | 57.9 ± 76.7 | 54.2 ± 70.9 | 0.180 | 25–35 | 46% | 10% | 44% |
| Milk and its products | 13.4 ± 52.8 [#] | 12.2 ± 57.8 | 10.2 ± 42.6 | 9.0 ± 39.8 | 21.7 ± 63.2 | 0.389 | >300 | 100% | - | 0% |
| Oil | 45.8 ± 102.6 [*] | 44.8 ± 45.1 | 41.3 ± 34.2 | 49.3 ± 106.4 | 47.3 ± 155.8 | 0.420 | 25-30 | 33% | 9% | 57% |
| Salt | 9.9 ± 17.8 [*] | 11.0 ± 12.5 | 9.5 ± 9.3 | 9.8 ± 22.1 | 9.5 ± 22.2 | 0.241 | <6 | 35% | - | 65% |

Notes: [a] Values are presented in mean ± standard deviation; [*] Significantly greater than the upper bound of dietary guideline at $p < 0.05$; [#] Significantly smaller than the lower bound of dietary guideline at $p < 0.05$; [b] Percentage of observations whose real consumption fall under, within or above the recommend level of dietary guideline.
